# Internet Gaming Addiction in Children and Adolescents: A Narrative Review in the Context of Screen Time

**DOI:** 10.7759/cureus.113441

**Published:** 2026-07-27

**Authors:** Neena Sharma

**Affiliations:** 1 Department of Physiology, All India Institute of Medical Sciences (AIIMS), Jammu, Jammu, IND

**Keywords:** adolescents, gaming disorder, internet gaming addiction, pediatric mental health, screen time

## Abstract

Internet gaming addiction in children and adolescents is an emerging clinical and public health concern within the broader context of increasing recreational screen exposure. Despite growing recognition of gaming disorder, uncertainty remains regarding its distinction from high recreational gaming, its relationship with screen time, and the most appropriate prevention and management strategies for pediatric populations. This narrative review aimed to synthesise evidence published between 2013 and 2026 on diagnostic concepts, epidemiological patterns, screen-time context, mental and physical health associations, and intervention approaches for problematic gaming in children and adolescents. Relevant literature was reviewed and organised into diagnostic, clinical, public health, and therapeutic domains. The conclusions were derived from qualitative narrative synthesis rather than pooled quantitative analysis. Current evidence indicates that gaming disorder should not be defined by screen-time duration alone, but by impaired control, persistence despite harm, prioritisation of gaming over essential activities, and functional impairment across academic, social, developmental, and family domains. Prevalence estimates vary because of differences in diagnostic criteria, assessment tools, cultural gaming practices, and study populations. Excessive gaming commonly overlaps with emotional symptoms, sleep disturbance, sedentary behaviour, reduced physical activity, academic difficulties, social withdrawal, and family conflict. Available evidence suggests potential benefit from multidimensional management involving validated assessment, cognitive behavioural strategies, relapse prevention, parental involvement, school-based digital literacy, structured offline activities, and coordinated pediatric mental health care. Screen-time reduction is important, but restriction alone is insufficient. Given the absence of formal evidence grading and risk-of-bias assessment, conclusions regarding intervention effectiveness should be interpreted cautiously. Pediatric gaming disorder requires impairment-based assessment and developmentally appropriate, family-supported intervention rather than simple screen-time control.

## Introduction and background

Internet gaming addiction among children and adolescents is an emerging pediatric public health and mental health concern that requires balanced interpretation to avoid labelling intensive but non-disordered gaming as a clinical condition [1]. Its relevance has increased as online gaming, mobile-device access, and digitally mediated recreation have become embedded in the daily lives of children and adolescents. Internet gaming disorder (IGD) is included in the Diagnostic and Statistical Manual of Mental Disorders, Fifth Edition, Text Revision (DSM-5-TR) as a condition for further study [2]. Gaming disorder is formally classified in the World Health Organisation’s International Classification of Diseases, 11th Revision (ICD-11) and is characterised by impaired control over gaming, increasing priority given to gaming over other activities, and continuation or escalation despite negative consequences [3]. Under both frameworks, clinical concern is based primarily on persistent functional impairment rather than gaming duration alone [3,4].

High gaming participation may represent recreational, social, competitive, or stress-relieving activity and does not necessarily indicate a disorder [4]. Overpathologising common gaming behaviour may reduce diagnostic specificity, particularly among children and adolescents whose patterns of digital engagement are still developing. The DSM-5 framework assesses features such as preoccupation, withdrawal-like symptoms, tolerance, unsuccessful attempts to reduce gaming, loss of interest in other activities, continued gaming despite problems, deception, escape-related gaming, and associated functional consequences [5]. The DSM-5-TR and ICD-11 frameworks overlap in their emphasis on impaired control and negative consequences but differ in their diagnostic structure, thresholds, and clinical application [6].

For consistency, this review uses “internet gaming disorder” for the DSM-5-TR construct, “gaming disorder” for the ICD-11 diagnosis, “problematic gaming” for clinically concerning behaviour that may not meet formal diagnostic thresholds, and “high recreational gaming” for intensive gaming without functional impairment.

Gaming disorder has been reported across countries and populations, although prevalence estimates vary according to diagnostic criteria, screening instruments, sampling methods, and cultural gaming practices [7]. Adolescents and young adults represent an important risk group, with reported associations involving male sex, greater gaming involvement, psychological distress, impulsivity, poor family functioning, and academic or social difficulties [8]. Differences between DSM-5- and ICD-11-based case definitions further limit direct comparison of prevalence estimates [9]. Longitudinal evidence suggests that gaming disorder may both contribute to and arise from psychosocial difficulties, including emotional symptoms, peer problems, academic stress, and maladaptive coping [10]. Problematic gaming may also persist into young adulthood, supporting the importance of early identification during childhood and adolescence [11].

Reliable assessment is essential because time spent gaming does not, by itself, distinguish high engagement from pathological gaming [12]. The Internet Gaming Disorder Scale provides a structured assessment of DSM-5-related symptom patterns in younger populations [12]. Population-based findings indicate that symptom clustering and functional impairment are more diagnostically informative than exposure duration alone [13]. Interpretation remains difficult because studies differ in their definitions, instruments, populations, and follow-up periods [14].

Screen time provides a broader developmental and public health context but is not synonymous with gaming disorder [15]. General screen exposure among children and adolescents has been associated with sleep disturbance, sedentary behaviour, overweight, emotional symptoms, and reduced physical activity [15]. Gaming disorder differs from general screen exposure through persistent loss of control, prioritisation of gaming, continuation despite harm, and impairment in daily functioning [3].

Existing reviews have commonly examined diagnostic frameworks, prevalence, screen exposure, health outcomes, or treatment approaches as separate topics. Limited synthesis has integrated gaming-specific impairment with broader paediatric evidence concerning mental health, sleep, physical activity, family functioning, school performance, and recreational screen exposure. This separation complicates the distinction between intensive recreational gaming and clinically impairing gaming disorder and limits translation into developmentally appropriate care. A narrative review is suitable for integrating heterogeneous evidence across diagnostic, epidemiological, clinical, preventive, and therapeutic domains. Important evidence gaps include inconsistent severity thresholds, heterogeneous assessment tools, limited longitudinal paediatric data, variable application of DSM-5 and ICD-11 criteria, inadequate cultural diversity, and insufficient examination of parental mediation, psychiatric comorbidity, digital competence, sedentary behaviour, and school-related factors [6,8,10,12].

Objectives of the review

This narrative review examines the diagnostic concepts, epidemiological patterns, screen-time context, health associations, risk and protective factors, and prevention and management of gaming disorder among children and adolescents. It also identifies evidence gaps relevant to paediatric practice, mental health services, families, schools, and public health policy.

Literature search methodology

A narrative literature search was conducted using PubMed, Scopus, Web of Science, and Google Scholar to identify relevant publications from January 2013 to June 2026. The search strategy combined terms related to the study population, gaming-related disorders, screen exposure, clinical outcomes, and interventions. Search terms included “internet gaming disorder,” “gaming disorder,” “internet gaming addiction,” “problematic gaming,” “children,” “adolescents,” “screen time,” “mental health,” “sleep,” “sedentary behaviour,” “cognitive behavioural therapy,” “relapse prevention,” “family intervention,” “parent intervention,” “school-based intervention,” “digital literacy,” “mindfulness,” and “yoga.” Boolean operators such as “AND” and “OR” were used according to the requirements of each database. The core search string was: (“internet gaming disorder” OR “gaming disorder” OR “internet gaming addiction” OR “problematic gaming”) AND (child OR adolescent OR pediatric OR paediatric) AND (“screen time” OR “mental health” OR sleep OR “sedentary behaviour” OR intervention OR treatment OR prevention OR “cognitive behavioural therapy” OR family OR school OR mindfulness OR yoga). This strategy was adapted to the syntax and indexing requirements of each database.

English-language original studies, randomised controlled trials, observational studies, systematic reviews, meta-analyses, and relevant clinical or public health literature addressing gaming disorder, problematic gaming, screen exposure, associated health outcomes, prevention, or treatment among children and adolescents were considered. When several publications addressed the same topic, systematic reviews and meta-analyses were prioritised, followed by randomised controlled trials, longitudinal studies, and relevant observational studies. Individual studies were retained when they provided recent, paediatric-specific, culturally relevant, or insufficiently synthesised evidence. Emerging intervention studies were included only when they addressed an area with limited completed paediatric evidence and were interpreted cautiously. Titles and abstracts were screened by the author, followed by full-text assessment of potentially relevant publications. Eligibility was determined according to the predefined inclusion and exclusion criteria.

Studies focusing exclusively on adults, articles unrelated to gaming or paediatric screen exposure, non-peer-reviewed commentaries, and publications lacking sufficient clinical or methodological information were excluded. One relevant preprint was retained because it examined a recent intervention within the broader paediatric screen-time context; its preliminary status was considered during interpretation. Reference lists of relevant articles were also examined to identify additional publications. Broader studies concerning paediatric screen time, smartphone use, obesity, and digital behaviour were included only as contextual evidence and were distinguished from gaming-disorder-specific findings.

The evidence was synthesised narratively and organised into diagnostic, epidemiological, clinical, preventive, and therapeutic domains. A narrative review design was selected because the literature was heterogeneous in diagnostic frameworks, populations, interventions, outcomes, and study designs, and because the review aimed to integrate gaming-specific evidence with the broader paediatric screen-time literature. No formal meta-analysis was conducted because of substantial heterogeneity in diagnostic definitions, study populations, interventions, and outcome measures. The conclusions were derived from qualitative narrative synthesis rather than pooled quantitative evidence integration. The synthesis compared convergent and divergent findings and considered methodological limitations, including small or selected samples, self-reported outcomes, short follow-up periods, cultural variability, and inconsistent diagnostic thresholds. Greater interpretive weight was assigned to systematic reviews, meta-analyses, randomised controlled trials, and longitudinal studies. Cross-sectional studies, protocols, and preliminary evidence were used mainly to provide contextual or emerging evidence and were interpreted cautiously.

Because this article is a narrative review rather than a systematic review, Preferred Reporting Items for Systematic Reviews and Meta-Analyses (PRISMA)-based study-selection reporting was not applied. No formal study-level quality appraisal or risk-of-bias tool was used. Methodological features such as study design, sample size, follow-up duration, outcome measurement, and generalisability were considered during interpretation. The absence of duplicate screening, formal risk-of-bias assessment, and quantitative evidence grading limits reproducibility and may increase susceptibility to selection and interpretation bias. Accordingly, conclusions concerning intervention effectiveness should be interpreted cautiously and should not be regarded as pooled or formally graded estimates of treatment effect.

The DSM-5-TR and ICD-11 were consulted as primary sources for the diagnostic classification of internet gaming disorder and gaming disorder, respectively. Only brief, appropriately cited paraphrased descriptions of these frameworks were included. The manuscript does not reproduce diagnostic criteria, tables, figures, assessment instruments, coding structures, or substantial verbatim content from either source. Their use was limited to descriptive discussion within this narrative review.

## Review

Diagnostic frameworks and clinical recognition of gaming disorder

Clinically impairing gaming should be distinguished from high but non-pathological digital engagement. Longitudinal evidence indicates that the relationship between screen exposure and adverse outcomes is complex and cannot be explained by duration alone, as emotional symptoms, sleep disturbance, family context, and baseline psychosocial difficulties may influence both screen use and subsequent outcomes [16]. Systematic evidence in adolescents similarly shows that associations with mental health vary according to study design, outcome definition, and the type of digital activity assessed [17]. Clinical assessment should prioritise impaired control, persistence despite harm, functional impairment, academic decline, social withdrawal, and caregiver conflict rather than screen-time duration alone.

Structured cognitive behavioural prevention may reduce symptoms and progression of gaming disorder and unspecified internet-use disorder among adolescents, supporting early identification and intervention [18]. Increased digital exposure during the COVID-19 pandemic further demonstrated that prolonged screen use does not necessarily indicate gaming disorder [19]. Diagnosis should therefore consider symptom persistence, developmental context, and impairment across family, school, and social settings. The clinical distinction between high recreational gaming, problematic gaming, internet gaming disorder, and gaming disorder is summarised in Table 1.

**Table 1:** Clinical distinction between gaming patterns and gaming disorder DSM-5-TR: Diagnostic and Statistical Manual of Mental Disorders, Fifth Edition, Text Revision; ICD-11: International Classification of Diseases, 11th Revision

Construct	Framework or status	Core features	Role of functional impairment	Role of gaming duration	Clinical interpretation
High recreational gaming	Non-diagnostic pattern	Frequent or prolonged gaming for recreation, competition, social interaction, or stress relief	Absent	May be high	Does not constitute a disorder when control and daily functioning are preserved
Problematic gaming	Clinically concerning but not necessarily diagnostic	Emerging loss of control, conflict, emotional dysregulation, or disruption of routines	Present to varying degrees	May be elevated but is not independently diagnostic	Requires assessment and monitoring; formal diagnostic criteria may not be met
Internet gaming disorder	DSM-5-TR condition for further study	Preoccupation, withdrawal-like symptoms, tolerance, unsuccessful attempts to reduce gaming, loss of interest, continued gaming despite harm, deception, escape-related gaming, and functional consequences	Required for clinical significance	Insufficient alone	Diagnosis depends on symptom clustering, persistence, and clinically significant impairment
Gaming disorder	ICD-11 diagnosis	Impaired control, increasing priority given to gaming, and continuation or escalation despite negative consequences	Central diagnostic requirement	Insufficient alone	Persistent behavioural pattern causing significant impairment in personal, family, social, educational, or occupational functioning

Screen time, mental health, and behavioural dysregulation

Problematic gaming becomes clinically relevant when it co-occurs with emotional distress, impaired self-regulation, and functional difficulties. Reported associations include anxiety, depressive symptoms, irritability, sleep disturbance, academic difficulties, social isolation, and family conflict [20]. Children and adolescents may also continue gaming despite adverse consequences and experience impairment across home, school, and peer settings [21].

Psychological interventions targeting maladaptive cognitions, emotional regulation, motivation, coping, and daily routines may reduce problematic gaming symptoms [22]. Cognitive behavioural therapy is among the most frequently studied approaches and may improve addictive symptoms by addressing maladaptive beliefs, craving, avoidance coping, and impaired self-control [23]. Treatment effects remain variable because studies differ in age, baseline severity, psychiatric comorbidity, intervention intensity, outcome measures, and follow-up duration.

Screen-time reduction and paediatric public health relevance

Excessive recreational screen exposure is associated with poor sleep hygiene, physical inactivity, sedentary behaviour, and reduced daily functioning. Structured behavioural interventions may reduce paediatric screen exposure, particularly when parental participation and routine modification are included [24]. Multicomponent interventions may also reduce broader internet-addiction symptoms, although their content and outcome measures vary [25].

Digital competence may help children identify online risks, regulate gaming behaviour, and develop healthier digital-use practices [26]. Relapse-prevention models emphasise emotional triggers, high-risk situations, ineffective coping, family communication, and recurrence after improvement [27]. Screen-time reduction should therefore form part of a broader strategy involving sleep hygiene, physical activity, parental monitoring, school functioning, and digital literacy.

Cognitive behavioural and relapse-prevention interventions

Cognitive behavioural and relapse-prevention approaches target the cognitive, emotional, behavioural, and environmental factors associated with problematic gaming. prevention frameworks support psychoeducation, risk recognition, behavioural regulation, and proportionate policy responses rather than restrictive measures alone [28]. Relapse-prevention therapy may address craving, emotional triggers, avoidance coping, peer influence, and high-risk situations among children and adolescents receiving psychiatric care [29].

Comparative evidence indicates that treatment effects differ according to therapeutic model, participant age, baseline severity, family participation, and clinical setting [30]. Family-centred models address parent-child conflict, inconsistent household rules, communication difficulties, and excessive screen use [31]. No single intervention has been established as universally superior, supporting an individualised approach based on developmental stage, comorbidity, family context, and functional impairment.

School-based and early preventive strategies

School-based prevention is relevant because problematic digital-use patterns may develop before children reach clinical services. structured programs can reduce problematic digital technology use and screen exposure, supporting schools as settings for digital literacy and early behavioural monitoring [32]. Preventive interventions in younger children also support age-appropriate education, parental guidance, and healthy offline activities [33].

Gaming-related difficulties may coexist with emotional symptoms, poor self-regulation, sleep disturbance, family problems, and broader digital overuse [34]. Intervention models include psychoeducation, behavioural regulation, parental monitoring, self-control training, and technology-supported strategies [35]. Evidence specific to formally diagnosed gaming disorder remains more limited than evidence for general digital overuse, and long-term effects are not yet established consistently.

Physical health, sedentary behaviour, and obesity context

Prolonged sedentary digital activity may displace physical activity, outdoor play, and sleep. screen-reduction interventions may contribute to improvements in weight-related outcomes among children and adolescents [36]. Mobile health education may support self-monitoring, goal setting, behavioural regulation, and healthier digital routines [37].

Associations among leisure screen time, internet gaming disorder, and mental health symptoms support integrated assessment of emotional wellbeing, sleep quality, physical inactivity, and daily functioning [38]. Mindfulness-based cognitive approaches are also being evaluated for adolescents with anxiety, depression, impulsivity, and problematic internet use [39]. These findings support holistic assessment but do not establish that gaming disorder independently causes obesity or other physical-health outcomes.

Complementary, family-based, and parent-focused approaches

Problematic gaming may be influenced by self-regulation difficulties, stress-coping patterns, family dynamics, and caregiver-child communication. An eight-week yoga intervention among adolescents with internet gaming disorder reported improvements in emotional regulation, attention, self-discipline, and offline engagement [40]. Early digital health education may also help prevent excessive screen exposure from becoming embedded in family routines [41].

Non-pharmacological interventions, including behavioural, psychological, family-based, and activity-oriented approaches, may reduce youth internet-addiction symptoms [42]. Parent-focused interventions further support monitoring, consistent boundaries, communication, and healthy digital modelling [43]. Family involvement appears clinically relevant, but the evidence remains heterogeneous and includes broader internet and smartphone-use outcomes rather than gaming disorder alone.

Digital interventions, therapeutic innovation, and integrated care models

Psychological interventions delivered across digital and conventional formats may reduce problematic use of the internet, video games, social media, and instant messaging, although effects vary according to platform, symptom severity, treatment format, and follow-up duration [44]. Parents and therapists have identified practical barriers involving engagement, caregiver burden, privacy, child resistance, and age-appropriate content [45].

Self-determination theory-based approaches seek to reduce unhealthy screen use by strengthening autonomy, competence, intrinsic motivation, and participation in alternative activities [46]. Integrated care may combine psychological treatment, family participation, school involvement, digital literacy, structured offline activity, and paediatric mental health follow-up. Its effectiveness requires further evaluation across age groups, cultures, and diagnostic frameworks. The principal prevention and management approaches, their targets, benefits, and limitations are presented in Table 2.

**Table 2:** Prevention and management approaches for problematic gaming in children and adolescents

Approach	Primary target	Main components	Evidence base	Potential benefits	Principal limitations
Cognitive behavioural therapy	Maladaptive beliefs, craving, impaired self-control, and avoidance coping	Cognitive restructuring, self-monitoring, coping skills, emotional regulation, and routine modification	Randomised trials, systematic reviews, and meta-analyses	May reduce addictive symptoms and functional impairment	Effects vary by age, baseline severity, treatment intensity, and follow-up duration
Relapse-prevention therapy	Recurrence after initial symptom improvement	Trigger identification, craving management, high-risk situation planning, and coping skills	Randomised and emerging clinical evidence	May support treatment maintenance and reduce recurrence	Limited long-term paediatric evidence
Family-centred intervention	Parent-child conflict, inconsistent rules, and household gaming patterns	Family communication, consistent boundaries, parental monitoring, and shared routines	Feasibility, clinical, and parent-focused studies	May improve adherence, communication, and home-based regulation	Heterogeneous intervention formats and limited large-scale trials
School-based prevention	Early problematic digital-use patterns	Digital literacy, psychoeducation, behavioural monitoring, and teacher-parent collaboration	Systematic reviews and school-based studies	May reduce problematic digital use and support early identification	Evidence often concerns general digital overuse rather than diagnosed gaming disorder
Digital competence education	Risk recognition and healthy technology use	Media literacy, self-regulation, critical evaluation, and balanced digital habits	Observational and preventive evidence	May strengthen protective digital-use skills	Causal and long-term evidence remains limited
Screen-time reduction	Excessive recreational screen exposure and sedentary behaviour	Routine modification, parental participation, device boundaries, and offline activities	Randomised trials and meta-analyses	May improve daily routines, physical activity, sleep, and weight-related outcomes	Screen-time reduction alone does not address compulsivity or functional impairment
Mindfulness and yoga	Emotional dysregulation, impulsivity, anxiety, and poor attention	Mindfulness practice, breathing exercises, attention training, and structured physical activity	Emerging trial evidence	May improve emotional regulation, attention, and offline engagement	Small or setting-specific studies and limited replication
Mobile and digital interventions	Self-monitoring, engagement, and behavioural regulation	Goal setting, digital education, progress monitoring, and motivational support	Emerging intervention and qualitative evidence	May increase accessibility and engagement	Privacy concerns, caregiver burden, resistance, and uncertain long-term effectiveness
Integrated care	Multidimensional impairment across home, school, and mental health domains	Psychological treatment, family participation, school involvement, digital literacy, and paediatric follow-up	Conceptual and mixed intervention evidence	Addresses clinical, developmental, and environmental factors together	Comparative effectiveness has not been established consistently

Limitations and future directions

The literature remains heterogeneous in diagnostic definitions, participant age, gaming format, assessment instruments, intervention design, and outcome measures. DSM-5 internet gaming disorder and ICD-11 gaming disorder overlap but are not equivalent, affecting prevalence estimates and clinical interpretation. Many studies rely on self-reported gaming behaviour and screen exposure, creating potential recall and social-desirability bias.

Several studies examine general screen exposure, internet addiction, smartphone use, obesity, or digital overuse rather than gaming disorder specifically. These findings provide developmental and public health context but should not be interpreted as direct evidence of gaming-disorder-specific effects. Selected samples, short follow-up periods, cultural variability, inconsistent diagnostic thresholds, and heterogeneous interventions limit firm conclusions regarding comparative effectiveness.

As a narrative review, this study did not use duplicate screening, formal study-level quality appraisal, risk-of-bias assessment, quantitative evidence grading, or PRISMA-based study-selection reporting. Although study design, sample size, follow-up duration, outcome measurement, and generalisability were considered during interpretation, the absence of formal appraisal methods limits reproducibility and may increase susceptibility to selection and interpretation bias. The conclusions are based on qualitative narrative synthesis rather than pooled quantitative evidence integration. Because certainty of evidence was not formally graded, conclusions regarding comparative effectiveness should be considered provisional.

Future studies should use standardised diagnostic criteria, validated paediatric instruments, larger samples, and longitudinal designs. Research should distinguish recreational, social, competitive, mobile, and pathological gaming and evaluate treatment maintenance, relapse prevention, family-centred care, digital interventions, mindfulness-based approaches, and school-based prevention.

## Conclusions

Gaming disorder in children and adolescents is defined by impaired control, continued gaming despite harm, and clinically significant disruption of academic, social, family, or developmental functioning rather than screen-time duration alone. High recreational gaming should therefore be distinguished from problematic gaming and formally diagnosed as a disorder through impairment-based assessment. Effective management should combine early recognition, validated assessment, cognitive behavioural and relapse-prevention strategies, parental involvement, school-based digital literacy, structured offline activities, and coordinated pediatric mental health support. Broader screen-time reduction may support sleep, physical activity, and daily functioning but should not replace assessment of motivation, coping, comorbidity, and family context. Future research should improve diagnostic consistency, longitudinal evidence, cultural representation, and long-term treatment follow-up.
